# Anticoagulation‐refractory strokes and selective infarction pattern: What’s the link?

**DOI:** 10.1002/ccr3.3502

**Published:** 2020-12-08

**Authors:** Alexandru Dimancea, Amalia Ene, Raluca Badea, Athena Ribigan

**Affiliations:** ^1^ Neurology Department Bucharest University Emergency Hospital Bucharest Romania

**Keywords:** aortic arch, case report, complex aortic plaque, dual antiplatelet therapy, ischemic stroke, vertebral artery

## Abstract

Stroke etiology in the form of a CAP should be actively explored, especially in the context of previous negative work‐up and anticoagulant‐refractory strokes. Dual antiplatelet therapy proved superior to anticoagulation for secondary prevention.

## Graphical abstract

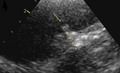

## 1 INTRODUCTION

Severe aortic arch atherosclerosis has been increasingly recognized over the past three decades as a risk factor and mechanism for ischemic stroke. Complex aortic plaques (CAPs) are defined in the majority of studies as having a thickness of over 4 mm or a superimposed mobile component (thrombus).^1^, ^2^, ^3^ The rate of recurrent ischemic stroke is especially high in patients with stroke and CAP (especially with an overlying thrombus).^1^, ^2^, ^4^

Embolization from CAPs occurs frequently into the left cerebral circulation.^1^ Transesophageal echocardiography (TEE) remains the gold standard for evaluating the aortic arch.^1^, ^3^ Prevalence of CAPs in stroke patients was estimated between 14% and 21%, and up to half of CAPs have a mobile component.^1^

Antithrombotic treatment in CAPs is subjected to controversy. The Aortic Arch Related Cerebral Hazard (ARCH) Trial suggested that dual antiplatelet therapy strategy might be preferred in clinical practice.^5^

## 2 CASE REPORT

We report the case of a 70‐year‐old male patient, with a history of smoking, arterial hypertension, dyslipidemia, and diabetes mellitus type 2, treated with metformin, indapamide, and amlodipine, and admitted for two sudden episodes of horizontal nystagmus accompanied by nausea and vomiting. Initial CT scan, ultrasonography of the cervico‐cerebral vessels, and transthoracic echocardiography were unrevealing; however, one episode of paroxysmal atrial fibrillation (AF) was detected on admission; a probable diagnosis of acute ischemic stroke of cardioembolic etiology (nonvalvular AF with a CHA_2_DS_2_‐VASc score of 5 points) was established, the patient being initiated on apixaban and statin. No further periods of AF were detected on a 24‐hour ECG Holter monitorization and further daily ECG studies.

During hospitalization, the patient developed sudden left ear neurosensorial deafness, followed several hours later by left hemiataxia, despite proper anticoagulation; emergency CT scan demonstrated multiple hypodense areas in the left cerebellar hemisphere; apixaban was switched to intravenous unfractionated heparin (UFH) therapy.

**Figure 1:**
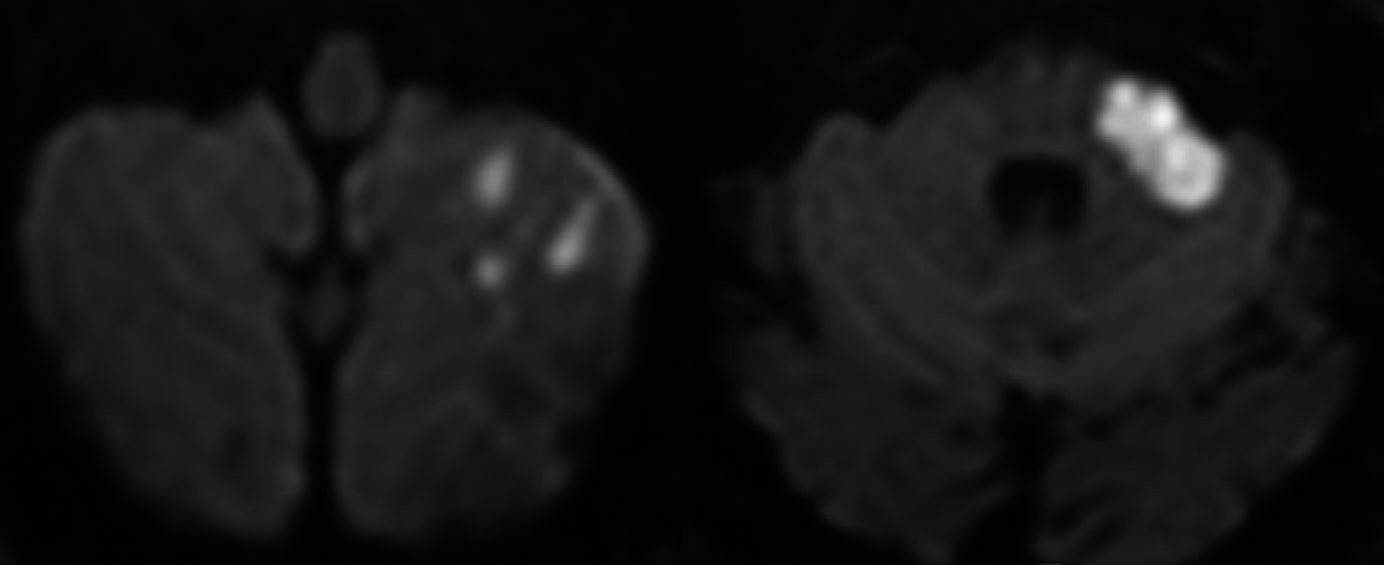
MRI‐DWI showing multiple small areas of restricted diffusion in the inferior left cerebellar hemisphere, and at the level of left middle cerebellar peduncle, also present on fluid attenuated inversion recovery sequence (not shown), suggesting subacute ischemic lesions

An MRI performed the next day revealed the aforementioned cerebellar infarctions along with subacute infarction of the left middle cerebellar peduncle (MCP), extending into the left lateral pons (Figure 1). CT angiography (CTA) revealed a left vertebral artery (VA) emerging directly from the aortic arch, with an atherosclerotic plaque near its origin (not shown). Aspirin was added to the previous treatment. After several days without neurological events, the patient was switched from unfractionated heparin to dabigatran, with maintenance of aspirin.

The presence of patent foramen ovale was investigated by means of transcranial Doppler ultrasonography at the level of the middle cerebral arteries and injection of contrast solution; however, no high‐intensity transient signals were recorded; furthermore, the recording was continued for thirty minutes, but no spontaneous micro‐embolic signals were detected.

During the end of the hospital stay, the patient abruptly developed left peripheral facial palsy, considered to be a result of infarction extension.

**Figure 2:**
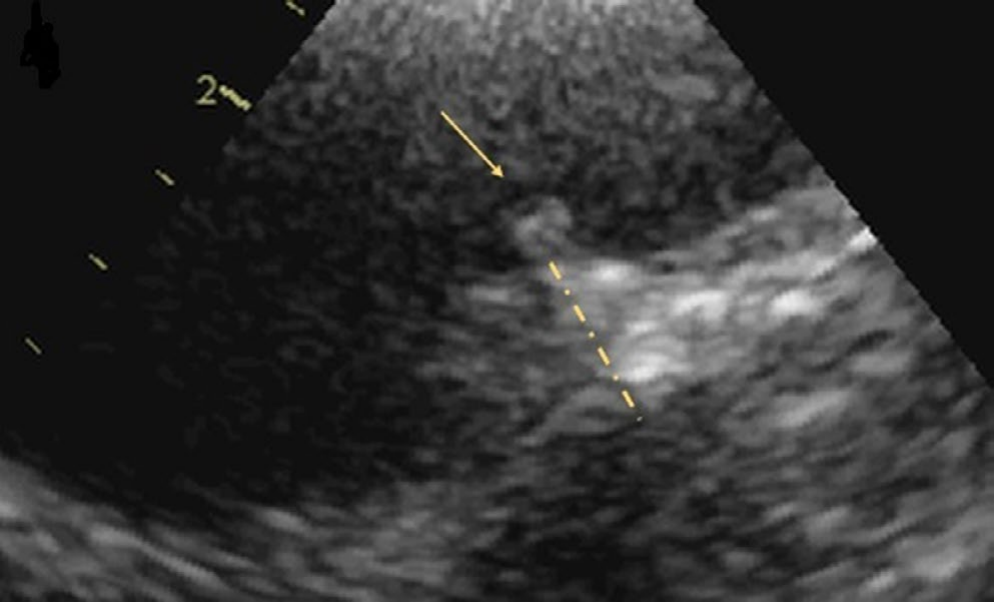
TEE revealing an aortic arch plaque with a maximum thickness of 9 mm (dotted yellow line) with a superimposed mobile component (yellow arrow)

TEE was performed, being mandated by inconclusive previous investigations and unsuccessful recurrence control. A complex, ulcerated atherosclerotic plaque with a mobile component, with a measured, maximum thickness of 9 mm was identified (Figure 2, arrow). Furthermore, no thrombus or dense spontaneous echo contrast was identified at the level of the left atrial appendage. Clopidogrel was thus finally added to treatment.

Except for deafness, the patient recovered completely and was discharged on dabigatran (a HAS BLED score of 3 for age, stroke history, and concomitant antiplatelet therapy), dual antiplatelet therapy (for one month, then aspirin alone), and maximum dose statin (40 mg rosuvastatin), with no ischemic recurrence since, to our knowledge.

## 3 DISCUSSION

CAPs are viewed both as a risk factor and mechanism of ischemic stroke: risk factor, through its association with classical cardiovascular risk factors^1^, ^3^ and carotid stenosis,^1^ as a marker of generalized atherosclerosis; probable mechanism, being associated retrospectively with stroke occurrence and recurrence. In a cornerstone autopsy study, examining 500 patients with cerebrovascular and other neurological diseases, ulcerated plaques were discovered in 26% of stroke patients and in only 5% of patients dying from other diseases.^6^ In another retrospective case‐control study, plaques thicker than 4 mm were associated with a 9‐fold increased odds ratio for cerebral infarction.^2^

Regarding infarction distribution, the left VA (or posteroinferior cerebellar) and anteroinferior cerebellar arteries territories were involved. This selective, unilateral, subtentorial distribution was unusual for a cardioembolic etiology. However, the presence of a nonvalvular paroxysmal AF required administration of a nonvitaminic K oral anticoagulant as preferred first‐line antithrombotic therapy.^7^

The first ischemic recurrence was followed by apixaban discontinuation and by switch to UFH, which permitted strict laboratory monitorization of anticoagulation efficiency. The possibility of treatment resistance was taken into consideration, as recurrence under apixaban was present in 1.27%/year in patients enrolled in the ARISTOTLE (Apixaban vs Warfarin in Patients with Atrial Fibrillation) Trial.^8^ UFH discontinuation was followed by introduction of dabigatran.

Moreover, the presence of an intracardiac thrombus was considered. As the left atrial appendage (LAA) is the principal structure involved in the formation of thrombi in nonvalvular AF,^9^ it was thoroughly explored during TEE; however, no thrombus or spontaneous echo contrast was identified within the LAA and no specific non‐chicken wing morphology (which is otherwise considered as increasing the risk of ischemic stroke^10^) was described.

Consequently, AF under appropriate anticoagulation was unlikely to be responsible for recurrent ischemic strokes in our patient and a percutaneous LAA occlusion procedure was therefore not proposed. Aspirin co‐administration was motivated by the presence of important aortic arch atheromatous plaques on CTA. Retrospectively, apixaban treatment failure was probably related to the underlying arterial thromboembolic stroke etiology, rather than cardioembolic.

Regarding final anticoagulant treatment choice, dabigatran was preferred, owing to its different mechanism of action. Oral vitamin K antagonists were also considered. However, besides several historical disadvantages (such as therapy monitorization and patient compliance), anticoagulation with coumarins might interact with clopidogrel metabolism. In a cross‐sectional observational study in coronary artery disease patients under dual antiplatelet therapy with aspirin and clopidogrel, concomitant treatment with phenprocoumon (coumarin derivative) significantly attenuated in vivo antiplatelet effects of clopidogrel.^11^ Both drugs are metabolized by the cytochrome P450 hepatic enzyme system, sharing the principal two isoenzymes, involved in the excretion of coumarins and activation of clopidogrel (CYP2C9 and CYP3A4). Furthermore, dabigatran pharmacodynamics do not involve the hepatic microsomal system, being excreted predominantly by renal clearance.^12^, ^13^

Direct aortic arch emergence of the left VA constitutes the most common anatomic variant, with an estimated prevalence of 2.4%‐5.8%^14^ in the general population. In our case, the CAP was situated in the proximity of the ostium of the left VA.

The aforementioned anatomic and clinical particularities revealed CAP as the most probable stroke etiology. While overlying thrombi embolization was regarded as the main stroke mechanism, it might have additionally provided the “finishing touch” to otherwise stenosed cerebellar vessels.

To this day, no consensus has been reached regarding the optimal secondary prevention therapy in patients with CAP and ischemic stroke. The results of the ARCH Trial, comparing clopidogrel plus aspirin vs warfarin in this patient population, demonstrated a reduction of vascular events following an index stroke in patients on dual antiplatelet therapy, but this result lacked statistical significance. Moreover, dual antiplatelet therapy was correlated with a reduced rate of vascular death vs warfarin.^5^ Consequently, it was suggested as a preferred secondary prevention strategy in clinical practice, also employed in our case.

## 4 CONCLUSION

Stroke etiology in the form of a CAP should be actively explored, especially in the context of previous negative etiological work‐up and anticoagulant‐refractory recurrent strokes. In our case, the presence of a strategic CAP and an anatomic left VA variant has probably concurred to generate a particular stroke pattern. Optimal secondary prevention is still subject to debate, dual antiplatelet therapy proving slightly superior to anticoagulation. Thus, our case highlights the importance of future studies regarding the optimal treatment of this condition.

## 5 ETHICS STATEMENT

Signed consent was obtained from the patient for the purpose of writing this case report and is available upon request. In accordance with the patient's right to privacy, we state that no identifying information was included in the manuscript.

## CONFLICT OF INTEREST

None declared.

## AUTHOR CONTRIBUTIONS

All authors participated in the health care of the patient. The corresponding author performed the literature search and defined the medical context for the clinical case. All authors participated equally in writing the case report manuscript.

## Data Availability

The authors confirm that the data supporting the findings of this study are available within the article and its supplementary materials.
